# Placing intersectional inequalities in health

**DOI:** 10.1016/j.healthplace.2022.102761

**Published:** 2022-04-06

**Authors:** Clare Bambra

**Affiliations:** * Population Health Sciences Institute, Faculty of Medical Sciences, Newcastle University, Sir James Spence Building, Royal Victoria Infirmary, Newcastle upon Tyne, NE1 4LP, United Kingdom

**Keywords:** Intersectionality, Health equity, Health disparities, Health inequalities, Geography, Gender, Ethnicity, Socio-economic status

## Abstract

Research into geographical inequalities in health has focused almost exclusively on examining the effects of area-level deprivation and has been largely framed through a compositional-contextual lens, their inter-relationship and the influence of vertical macro-economic and political/policy drivers. However, in the broader health inequalities field, intersectionality has recently emerged as a critical theoretical and methodical approach which examines the concurrent and interacting influences on health of multiple axes of inequality (such as socio-economic status, gender, race/ethnicity and sexuality or gender-identity). Simultaneously, social geography has been explicitly using intersectionality to analyse how mutually constitutive forms of social oppression interact and interrelate with place. This paper exploits the analytical space opened up by this ‘intersectional turn’ by outlining the benefits for research into geographical inequalities in health that can be achieved by taking a more explicit approach to intersectional inequalities. It argues that: (1) geographical research into health inequalities should more explicitly and widely apply an intersectional lens; and relatedly that, in turn, (2) place needs to be considered as an aspect of intersectionality and integrated into the wider intersectional inequalities in health literature. The paper summarises the evolution of theories of place and health inequalities and outlines intersectional theory and the work to date that has been undertaken to integrate this perspective into our understanding of health inequalities. Drawing on the social geography literature into place and intersectionality, the paper explores how this perspective is being used to enhance our understanding of place effects more generally – and how place itself can be considered as an element of intersectional inequalities. Drawing these different bodies of work together, the paper concludes by considering the implications for theories of geographical inequalities in health.

## 1 Introduction

There are significant geographical inequalities in health with a 2-year gap in life expectancy between the northern and southern regions of England and up to 9 years difference in life expectancy between English neighbourhoods (Bambra, 2016). Likewise, in the US city of New Orleans, there is a 25-year gap in life expectancy between rich and poor neighbourhoods (Schrecker and Bambra, 2015). These geographical patterns in health outcomes are also evident in Europe (Thomson et al., 2017) – for example, in Oslo, Norway, life expectancy varies by up to eight years between districts (Norwegian Institute of Public Health, 2018) – as well as globally (WHO, 2008; Victorra et al., 2017). There are also significant inequalities in health between men and women (with higher rates of morbidity amongst women, and higher mortality amongst men [Bambra et al., 2021]); between ethnic groups (e.g. COVID-19 death rates are three-times higher amongst Black Americans [Bambra et al., 2020); by migration status (including inequalities in access to health care [Gkiouleka et al., 2020]); and amongst other marginalised groups (e.g. higher rates of mental ill health amongst LGBTQ + groups, [Byne, 2014]).

Research into geographical inequalities in health has largely focused on examining the effects of area-level deprivation and has often been framed through a compositional-contextual lens, their inter-relationship (Cummings et al., 2007) and the influence of vertical macro-economic and political/policy drivers (Bambra et al., 2019). However, in the broader health inequalities field, intersectionality *“has [recently] emerged as a critical theoretical and methodical approach”* and there has been a push to take a more intersectional approach (Abrams et al., 2020: 113) by examining the concurrent and interacting influences on health of multiple axes of inequality (such as socio-economic status, gender, race/ethnicity and sexuality or gender-identity) and their syndemic interactions (Kapilashrami et al., 2015; Hill, 2015; Kline, 2020; Bambra et al., 2021a). Further, social geography has been using intersectionality to analyse how mutually constitutive forms of social oppression interact and interrelate with place (Hopkins, 2019).

This paper exploits the analytical space opened up by this ‘intersectional turn’ by outlining the benefits for research into geographical inequalities in health that can be achieved by taking a more explicit approach to intersectional inequalities. It argues that: (1) geographical research into health inequalities should more explicitly apply an intersectional lens; and relatedly that, in turn, (2) place needs to be considered as an aspect of intersectionality and – as such - integrated into the wider intersectional inequalities in health literature. The paper summarises the evolution of theories of place and health inequalities and outlines intersectional theory and the work to date that has been undertaken to integrate this perspective into our understanding of health inequalities. Drawing on the wider social geography literature into place and intersectionality, the paper explores how this perspective is being used to enhance our understanding of place effects more generally – and how place itself can be considered as an element of intersectional inequalities. Drawing these different bodies of work together, the paper concludes by considering the implications for theories of geographical inequalities in health.

### 1.1 Place and health inequalities

To date, the literature seeking to explain geographical inequalities in health has been dominated by the composition-context debate, its reconciliation via the relational (Cummings et al., 2007; Macintyre et al., 2002) and neo-materialist (Fox and Powell, 2021) perspectives and, more recently, the integration of political economy and institutional perspectives (Bambra et al., 2019; Beckfield et al., 2015). This section briefly summarises the evolution of these approaches.

The compositional view argues that who lives in a place determines its health outcomes. This focuses primarily on the influence of the behaviours (smoking, alcohol, physical activity, diet, drugs) and socio-economic (income, education, occupation) and demographic (e.g. ethnicity, gender) characteristics of the people living within a particular area (neighbourhood, city, region, country): that *poor people result in poor places* (Bambra, 2016). In doing so, it highlights the importance of individual-level factors above the wider context. In contrast, the contextual approach highlights that it is *what a place is like that matters for health*. Health differs by place because it is also shaped by the economic (e.g. poverty rates, unemployment rates, wages, and types of work and employment [Macintyre, 2007]), social (e.g. *opportunity structures* such as the services provided such as child care, transport, food availability or access to a health care services [Pearce et al., 2007] and *collective social functioning* e.g. community social cohesion, collective social capital or control [McGowan et al., 2020], place attachment [Hawe and Shiell, 2000] or place-based stigma [Halliday et al., 2021]), and physical (e.g. green space, waste facilities, brownfield land, air pollution or ‘environmental deprivation’ [Bambra et al., 2014; Pearce et al., 2010]) environment of the area: that *poor places lead to poor health* (Bambra, 2016).

The contextual and compositional explanations for how place relates to health are not mutually exclusive and to separate them is an over simplification and ignores the interactions between these two levels (Macintyre et al., 2002): the characteristics of individuals are influenced by the characteristics of the area. Both compositional and contextual factors contribute to the complex relationship between health and place – an ecosystem made up of people, systems and structures (Cummins et al., 2007). A *relational approach* should therefore be taken to understanding how compositional and contextual factors interact to produce geographical inequalities in health (Cummins et al., 2007) with place considered as unbounded, fluid, and dynamic. This leads to analyses which integrate individual compositional level factors with horizontal contextual factors. Further, Fox and Powell (2021) have argued that the traditional health geography approaches have tended to create an artificial dualism between the social and physical aspects of place and they instead propose a neo-materialist approach which considers places as socio-material assemblages of human and non-human materialities. Contextual approaches to health and place have often distinguished between physical (material e.g. built or natural environment) and cultural (e.g. spatial stigma, cultural norms) and social (e.g. access to services) aspects of place – often privileging one or the other in terms of analytical importance. The neo-materialist approach argues that the material and the socio-cultural aspects of place are *relational* and cannot be disentwined in terms of their health effects. In this way, the neo-materialist approach considers places and spaces as assemblages, in which locations interact with multiple other human and non-human materialities (Powell et al., 2020); that ‘health’ needs to be considered not as an individual attribute but relationally and micropolitically, in terms of the affective and relational engagements between bodies and the material world (Fox and Powell, 2021); and that social position itself should be considered not at the individual-level, but as a set of capacities, contingent upon place-based assemblages (ibid.). The relational and neo-materialist approaches have also opened up the literature on health and place to begin to consider the influence of vertical macro-level political, economic and institutional factors (Bambra et al., 2019).

This has since led to the development of a political economy approach to health and place (Bambra et al., 2019). Drawing on the wider political economy of health inequalities literature (e.g. Bambra et al., 2005; Beckfield, 2018), Bambra et al. (2019) recently asserted that there needs to be more focus in health geography on *“the influence of the macro political and economic, structural factors shaping places and their influence on population health outcomes”* (p37). Arguing that the relationship between health and place - and the health inequalities that exist between places - are to a large degree politically determined, they outlined how the influence of compositional and contextual factors (and their relational nature) on health are in turn shaped by macro-level structural determinants: politics, the economy, the (welfare) state, the organisation of work, and the structure of the labour market - with patterns of disease *“produced, literally and metaphorically, by the structures, values and priorities of political and economic systems … Health in equities are thus posited to arise from whatever is each society’s form of social inequality, defined in relation to power, property and privilege”* (Krieger, 2003: 430).

However, despite the nuances in our theoretical understanding of the relationship between health and place that this evolution has provided, all the resulting research (from whichever perspective) has often been limited to the examination of the relationship between place, health and a single axis of inequality (most notably area-level deprivation, and, to a lesser extent socio-economic status, ethnicity or migration [e.g. Bécares et al., 2013; Darlington-Pollock et al., 2017]) – with occasional reference to other factors such as gender (e.g. Rocha et al., 2017) or housing tenure (e.g. Darlington-Pollock and Norman, 2017). There has been little explicit integration into the geographies of health inequalities body of work of a more intersectional understanding of health inequalities and place - which explores how multiple axes of inequality are experienced simultaneously within – and as part of – a place. There are some notable recent examples (such as Evans, 2019a, 2019b or Bauer and Scheima, 2019) but these very much remain the exceptions, not the norm.

### 1.2 Intersectionality

Intersectionality was initially developed by Black feminist researchers and activists as a way to conceptualise the multiple disadvantage experienced by Black women (Crenshaw, 1991, 1989; Davis, 1983; hooks, 1981). Since then, intersectionality has influenced scholarship in various fields (including social geography, e.g. for an overview see Hopkins, 2019). Intersectionality considers that social categories (e. g. socio-economic status, gender, race, or sexuality) are mutually constructed and together lead to complex experiences of social inequalities. Inequalities vary historically, are culturally specific and vary across time and space (Gkiouleka et al., 2018). People are differentially located within a matrix of power, privilege and disadvantage (Yuval--Davis, 2015) – there is not a single, static social hierarchy in which one aspect of social position (e.g. social deprivation) is more important than another (Crenshaw, 1992). Different social categories (e.g. race, gender) interlink in shaping individual experiences – and health outcomes. Social groups therefore experience different amounts of disadvantage and privilege associated with their different characteristics – and related to their specific context (Nash, 2008). Groups might experience the benefits of privilege related to one system of power and stratification (e.g. advantage of whiteness in terms of race/ethnicity), whilst simultaneously engendering disadvantage of another (e.g. women in terms of gender roles) (Iyer et al., 2008; Nash, 2008).

This has led to the evolution of contrasting views within intersectionality theory around how multiple axes of inequality should be analysed: the *simultaneity* approach – argues that all axes of inequality must be investigated accumulatively in a study; the *multiplicativity* approach - that axes of inequality intersect to create complex social identities which are not merely a sum of their parts; and *multiple jeopardy* – that when disadvantaged identities are experienced simultaneously they tend to produce inordinate amounts of disadvantage (Abichahine and Veenstra, 2017). These different approaches are also reflected in McCall’s (2005) taxonomy of intersectional research approaches which distinguishes between anti-categorical (that social identities and contexts are too complex to be adequately categorised); inter-categorical (existing categories can be used to analyse inequalities, more typical of quantitative studies); and intra-categorical (examines those at the margins of categories, often the focus of qualitative research) intersectionality (Abichahine and Veenstra, 2017).

### 1.3 Intersectionality and health inequalities

The sizeable health inequalities literature has developed across quite independent streams but with a dominant (and arguably excluding) emphasis on socio-economic position as the key social determinant of health (Gkiouleka et al., 2018; Kapilashrami et al., 2015). Most health inequalities studies focus on single factors and mechanisms at a time – often even using single measures of socio-economic status (e.g. occupation *or* income *or* education). Similarly, research into racial/ethnic health inequalities, has often focused on the health disadvantage that members of minorities face due to their experience of discrimination (Nazroo and Williams, 2005). Immigration has also been treated as a distinct category in health inequalities research (Krieger, 2000). Like-wise, the gender and health inequalities literature has developed somewhat separately from the socio-economic and racial/ethnic literature (Bambra et al., 2021b). Sexuality and gender-identity research has also tended to be studied as autonomous from other dimensions of social difference (Gkiouleka et al., 2018) – and often only reported within specialist journals (e.g. see Byne, 2014).

This atomising approach in the health inequalities literature obscures the multiple stratification systems that people embody *simultaneously* (Krieger, 1997). The intersectional approach to health inequalities aims to address this by considering the cumulative, additive and integrated nature of health inequalities and the converging processes associated with different categories of disadvantage (Graham et al., 2011). As Barbeau et al. (2004: 273) have noted *“none of these social constructs is a stand-in for any other, and all are necessary for generating adequate depictions of social inequalities in health”*. Despite long standing calls to integrate intersectionality analyses (e.g. in 2003, Weber and Parra-Medina, argued that intersectionality provided a promising avenue for expanding knowledge of health disparities), it is only recently that health inequalities researchers have started to seriously undertake this process (Evans, 2019a, 2019b or Bauer and Scheima, 2019).

For example, Gkiouleka and Huijts (2020) integrated intersectionality theory to explore how health inequalities experienced by migrants in Europe interact with generation, occupational status and gender. They found that multiple relationships of health inequality operate simultaneously with the ‘healthy migrant effect’ seeming to apply most to first-generation immigrants working as manual employees, and that non-migrant women are more susceptible to poor self-rated health than migrant men. Similarly, King et al. (2019) examined differences in the effects of self-reliance on mental health between those with and without a disability in Australia. They found that men with disabilities who reported higher conformity to self-reliance norms had much worse mental health than non-disabled men with low conformity to self-reliance. Abichahine and Veenstra (2017) compared physical activity for Canadian men and women of different ethnicities, social classes, and sexual orientations. They found that the association between income and physical activity is stronger for ethnic minority men and lesbian, gay or bisexual women (but not men), than amongst White men and women or ethnic minority women. In another Canadian study, Ross et al. (2016) used mixed-methods to examine the interrelationships between bisexuality, poverty, and mental health. The quantitative component found that low-income bisexual participants had significantly higher rates of depression compared to higher income ones. The qualitative part of the study identified various potential pathways through which bisexuality, poverty and mental health intersect including early life experiences; sexual identity and employment; class and sexual orientation discrimination; and lack of access to mental health services. In a qualitative study utilising intersectional analysis conducted in Australia, Young (2020)examined health and wellbeing amongst African-Australians. She found that migration pathway, age, and gender were the systems of oppression/privilege that most impacted on health - operating through segregation and ‘othering’ in education, employment, and health care.

Example intersectional studies with a more geographical perspective are growing in number and include Alvarez and Evans (2021); Roy, Bhatta, and Burnette’s (2020); and Kapilashrami and Marsden (2018). Alvarez and Evans (2021) examined the role of gendered family structures, race/ethnicity, gender and deprivation in shaping inequalities in cancer risk from air toxins (Alvarez and Evans, 2021). They found that they are socially patterned across numerous intersecting axes of marginalisation. Roy, Bhatta, and Burnette (2020) examined the intersectional influences of education, gender, and region on later life functional health in India. They found that the effects of education on functional limitations were significantly greater for men than women but that this relationship varied regionally. Kapilashrami and Marsden (2018) used participatory methods to examine intersectional inequalities in access to health-enabling resources in disadvantaged communities in Scotland. They found that how the living environment affected access to resources varied with social location with, for example, notable differences in terms of race/ethnicity, gender, poverty and age. Whilst aspects of the physical environment (parks and places to worship) were common in both white and ethnic minority groups, very few community resources and public services (including health care) were seen as health enabling by ethnic minority women. Studies taking a more structural perspective have also examined intersectional inequalities in health and place. For example, Giesbrecht et al. (2018) used extensive ethnographic data to reveal how symbolic, aesthetic, and physical elements of formal healthcare ‘places’ intersect with social relations of power to produce, reinforce, and amplify structural vulnerability and inequities in access to care. Similarly, Sciarotta, and Hunter (2022) used participant observation and interviews to examine how the drug economy in Brazil interacts with and shapes social inequalities at the intersections of race, class, and gender in HIV transmission. Scott (2021) and Halliday et al. (2021) also used qualitative methods to examine the intersectional health impacts of experiences of stigma in low income neighbourhoods (Halliday et al., 2021) and amongst Black gay men in rural areas of the southern United States (Scott, 2021).

These example studies from across the health inequalities field begin to illustrate the benefits of applying an intersectional lens to health inequalities. However, the intersectional inequalities in health literature still remains small. By way of example, a recent systematic review of intersectional inequalities in mental health found only 20 studies and that *“few studies analysed factors potentially explaining the intersectional inequalities”* (Fagrell Trygg, Gustafsson and Månsdotter, 2019). Most notably for this paper, there has been very little integration of *place* itself as a facet of intersectionality in studies of health inequalities. The geographical health inequalities literature outlined previously, has also developed as an important (Gatrell and Elliot, 2009; Elliot, 2018) - but distinct and separate body of work, seldom integrated into wider studies of health inequalities (Bambra, 2016). As a result, its insights are currently lacking in the emergent literature on intersectional health inequalities. So, just as analyses of health and place have been limited to the exploration of single axes of inequality, the potential role of place as an aspect of social location and identity – differentially shaping health outcomes for otherwise similar social groups - has been absent from intersectional research into health inequalities. As the next section outlines, social geographers have started to explore how *place* shapes how the other social categorisations are experienced (Hopkins, 2019) – and we need to consider this in regards to geographical inequalities in health too.

### 1.4 Place and intersectionality

In a review article, Hopkins (2019) outlines how that from the late 1980s and early 1990s, a small group of social geographers started to explicitly integrate intersectional perspectives into the field (e.g. Peake, 1993; Kobayashi and Peake, 1994; Smith, 1990; Jackson, 1994; Ruddick, 1996). Since the 2000s, the intersectional geographies perspective has grown and become more prominent within the discipline (e.g. Valentine, 2007), engaging with diverse areas of geographical research (Hopkins, 2019). Crucially, in terms of the development of intersectional theory, social geographers have started to offer *place* – and locality specifically - as a crucial aspect of intersectionality (Hopkins, 2018). This has been done by examining the intersections of sexuality, class, race/ethnicity, age and sexuality with location (e.g. Rodó-de-Zarate, 2014; Schroeder, 2014) or exploring the intersectional nature of ethnic and religious identities, racism gender, social class and locality (Hopkins et al., 2017). However, Hopkins (2018) also argues that *‘the role of locality and place in shaping the intersections between different inequalities and power relations’* remains under-explored within social geography and that geographers have *‘yet to make a significant contribution’* to intersectionality (Hopkins, 2018: 586). As a result, *‘understanding and exploring the role that place, space and scale’* have for intersectionality is under-theorised (Hopkins, 2018: 586; Cho et al., 2013).

This is a clear gap for progressing our understandings of health and place given the importance of social context in intersectional studies. As Collins and Bilge (2016: 197) argue *‘social context has many interpretations’* and they point to the importance of historic context, states and their political and economic power, and social-cultural institutions as all contributing to ‘social context’ and localities. Indeed, Yuval-Davis (2015) describes intersectionality as a context informed analytical tool *(situated intersectionality)* that enables a focus on the social divisions shaping most people’s lives (e.g. race and gender) and how this plays out in different contexts (e.g. time, place). She argues that intersectionality is a theoretical lens that is sensitive enough to render visible other divisions shaping the experience of individuals and groups at marginal positions (e.g. sexuality) in different places. Individuals and groups are differentially located within the intersecting systems of power and their location – both in social and geographical terms - shaping their point of view of their own and others’ experience (Yuval-Davis, 2015).

However, beyond some applications within social geography, the potential of *situated intersectionality* - as a concept and analytical approach that explicitly takes into account the role of place, space and scale for different forms of inequality (Hopkins, 2019) – has been rather under-utilised by the health geography community specifically (for notable exceptions see Evans et al., 2019a, 2019b; Bauer and Scheim, 2019) – and by the wider health inequalities field in general (Bauer, 2014; Gkiouleka et al., 2018). So, just as health geographers need to take a more explicitly intersectional approach, the wider intersectionality and health inequalities field needs to consider the role that place plays as an aspect of intersectionality. Future studies need to take place, space and scale into account in studies of the intersectional nature of health inequalities. In doing so, health geographers can make a significant impact on both the broader health inequalities research community and the related fields of social geography and intersectionality (Hopkins, 2018).

### 1.5 Integrating intersectionality, place and health inequalities

The preceding sections have argued that geographical research into health inequalities should begin to more explicitly apply an intersectional lens; and that the wider intersectional inequalities in health literature also needs to consider place as an aspect of intersectionality. This section explores the implications of this call for theoretical development in the geographies of health inequalities field.

Intersectionality represents an exciting opportunity for the advancement of our conceptual understanding of geographical inequalities in health. However, integrating an intersectionality lens into geographical theories of health inequalities also poses a considerable challenge to some of the current perspectives in the field. On a superficial level, it could be quite easy to integrate intersectional perspectives into current theories of health and place - albeit in a rather reductionist, atheoretical manner (Evans, 2019a). For example, compositional approaches could be enhanced by the intersectional perspective by considering additional factors (beyond the traditional focus on social economic status, ethnicity, age, and health behaviours) and how they interact with one another. Similarly, contextual approaches could simply integrate an intersectional perspective by examining the impact on health of place (e.g. the economic, social opportunity structures, collective social functioning and physical environments) *“in shaping the meaning of particular intersectional social positions, the advantages or disadvantages they confer, the performance of those intersectional social identities, and/or the physical, social, economic, and political conditions individuals are exposed to, with short- and long-term implications for health”* (Evans, 2019a: 252). Relational and neo-materialist approaches could examine how an expanded, intersectional understanding of compositional factors interact with contextual factors to shape the health profiles of different places. But, as Evans (2019a) outlines, such descriptive intersectional studies would *“blunt [the] critical edge and transformative aims”* of intersectionality (May, 2015, p. 141).

A more comprehensive, theoretical reading of an intersectional lens is required to advance the field as *“engagement with theory is essential in order to maintain the critical and transformative edge of intersectionality”* (Evans, 2019a: 249). This poses a much more fundamental challenge to our existing theoretical literature on health and place and therefore requires a more substantial integration. Piecemeal attempts to integrate intersectionality will merely lead to descriptive, atheoretical, inter-categorical methods of data collection and analysis (Evans, 2019a) - which will ultimately only provide limited future empirical insights. To fully benefit from the transformative opportunities provided by an intersectional understanding of health and place, we need to radically rethink how place shapes health and to do this we need to take on board the most fundamental concept within the intersectionality lens – power (any relationship or arrangement whereby one group of persons is controlled by another [Millett, 1969]). Positions of privilege and disadvantage are not individual or group attributes but products of the power structures operating within the contexts in which we are embedded (Gkiouleka et al., 2018). The intersections between social categories are reflections of intersecting systems of power (Collins, 2015). Within an intersectional framing, the boundaries between structural context and individuals are fluid, permeable and interacting (Collins, 2015). This has implications for how we consider place effects on health: Individuals and their social identities are embedded within specific places where unique power dynamics and social processes are in effect (Gkiouleka et al., 2018). Thus intersectionality reframes health inequality in terms of power relations whereby certain groups enjoy a health privilege at the expense of others (Weber and Parra-Medina, 2003). This privilege is often embedded within institutions (Gkiouleka et al., 2018).

This framing has very strong implications for existing geographical theories of health inequalities as it reveals the limits of compositional, contextual and relational approaches - which are often too horizontal in their framing on place effects (*“overemphasising the role of lower level, localised, proximal contextual”* factors, [Bambra et al., 2019: 37]) and also have a bent towards intra-categorical, descriptive analysis (Evans, 2019a). What is needed therefore is a more vertical framing of place (*“the role played by larger scale contextual influences, particularly macro political and economic”* ones, [Bambra et al., 2019: 37]) that integrates intersectional understandings of power across multiple axes of inequality. The political economy approach has previously developed a more vertical and power focused framing of place (Bambra et al., 2019) – that is *“more explicitly critical and radical in orientation”* (Evans, 2019a: 250). However, to date, the political economy approach has focused almost exclusively on the implications of political and economic structures (such as labour markets, political institutions, and welfare regimes) for inequalities by socio-economic status only (Gkiouleka et al., 2018). Intersectionality shows that the next stage in the evolution of geographical theories of health inequalities requires the political economy approach to also incorporate the structural drivers of gendered and racialised inequalities in institutions such as patriarchy or structural-level racial discrimination (Stanistreet et al., 2005; Gkiouleka et al., 2018; Bauer and Scheim, 2019) – alongside those influencing socio-economic inequalities. Taking this more root and branch approach to integrating intersectionality will lead to a more nuanced understanding of the way in which ‘place’ (and power relations it represents) differentially influences the health of different groups of people.

The integration of intersectionality into theories of geographical inequalities in health also has implications for the methods of analysis used in the field. The different approaches to intersectionality (anti-categorical; inter-categorical; intra-categorical [McCall, 2005]) each lead to different foci (simultaneity; multiplicativity; multiple jeopardy) and correspondingly require different analytical techniques to be employed. The anti-categorical approach to intersectionality (in which social identities and contexts are considered to be too complex to be adequately categorised) has conventionally lent itself to more qualitative and ethnographic methods which enable research to simultaneously explore the accumulation of different aspects of identity (Abrams et al., 2020). However, the anti-categorical approach has also been integrated into quantitative studies of health inequalities through the statistical concept of discriminatory accuracy (Mulinari et al., 2018). This has led to wider calls for this type of research to be undertaken – alongside more conventional epidemiological inter-categorical analyses of between-group risk calculations (such as a odds ratios) – in order to overcome the high degree of outcome variability within, and overlap between, categories and to enable new insights into the processes underpinning the unequal distribution of health and wellbeing factors (Mulinari et al., 2018). Indeed, anti-categorical approaches can have strong implications for health interventions – adding more weight to the case for universal versus targeted approaches (Mulinari et al., 2018; Wemrell et al., 2021). has argued that this should be used to inform health inequalities research. The inter-categorical approach (in which existing categories can be used to analyse inequalities) has been the dominant approach to intersectionality in quantitative health inequalities research to date [Green et al. (2017). Example techniques which add a health geography angle include multilevel analyses of the contexts within which intersectional identities exist (e.g. schools, neighbourhoods, states) (Green et al., 2017; Evans, 2019b) as well as Structural Equation Modelling which would enable underlying processes such as racism or sexism to be captured by latent variables (Green et al., 2017). However, it should be noted that the ability of quantitative methods to capture intersectionality has been challenged by some scholars (Abrams et al., 2020). The intra-categorical approach (which examines those at the margins of categories) opens up the space for geographical inequalities in health research to examine the lived (and *placed*) experiences of more marginalised and socially excluded groups. For example, there is an emerging body of geographical work which examines how the experience of multiple forms of marginalisation in healthcare settings (Giesbrecht et al., 2018; Abrams et al., 2020).

This article has also argued that place needs to be considered as an aspect of intersectionality in future studies of health inequalities. Whilst the application of an intersectional perspective has already advanced the study of health inequalities (as summarised by Kapilashrami et al., 2015), using an intersectional lens to focus on individual social positioning alone is a necessary but not a sufficient way of developing an integrative understanding of health inequalities. We need to also consider how power, privilege and disadvantage are also shaped by - and experienced within - the different places and times (the contexts) - in which individuals are embedded. As outlined earlier, social iinequalities vary historically, are culturally specific and vary both temporally and spatially (Gkiouleka et al., 2018). Taking Yuval-Davis’ (2015) concept of *situated intersectionality*, we need to start to examine how social identities and their health implications play out differently across time, space and place. There are multiple examples of this but to give just a few, then we can think about how the mental health of LGBTQ + populations might vary over time (related to the historical evolution of gay and trans rights [Faderman, 2015]) and place (as a result of global and cultural differences in attitudes towards homosexuality, for example [Kite et al., 2018]); how gender inequalities in health vary across different countries (e.g. some countries have higher mortality rates amongst women - often related to maternal mortality, whilst in other countries it is male mortality that is higher [Bambra et al., 2021]); the changing socio-historical context of racial/ethnic inequalities in health (Gkiouleka et al., 2018); or how socio-economic inequalities in health differ between areas (e.g. inequalities in mortality by occupation are higher in the North East of England than in the South West of England [Marmot, 2010]). These examples are merely indicative of the varied ways in which place can shape how other social inequalities impact on health inequalities. Future health inequalities research - if conducted through an intersectionality and place lens - would highlight more, potentially benefitting public health practice.

Further, social geography has highlighted how place can itself be considered as an aspect of social identity and a basis for relationships of power, privilege and disadvantage (Hopkins, 2018). Most notably perhaps are examples of nationalism (e.g. evident in the “Brexit” vote for the UK to leave the European Union in 2016, the Scottish Independence Referendum of 2014, and the election of Donald Trump as US President in 2016) and the resulting ‘othering’ of non-native and migrant groups (Rash, 2012). Other examples include localism or regionalism (Paasi, 1986, 1991) and the role that place identity plays in terms of individual and group self-identification (Gieseking et al., 2014). It is already evident from extensive research within the health geography domain that place identification – and how places are viewed by the self and others – can have profound impacts particularly on mental health and wellbeing. Place-based stigma (or territorial/spatial stigma) is *“evident where a locality (*e.g. *a town, neighbourhood or housing estate/project) becomes marked out negatively on the basis of characteristics associated with the locality”* (Halliday et al., 2021: 1) such as racial segregation, high poverty rates or deindustrialization (Garthwaite and Bambra, 2018). This may lead to an area gaining a negative reputation in turn resulting in the embodiment of this by residents, with psychological and material impacts on residents’ life chances – even after they leave the area (Wacquant et al., 2014). Place-based stigma has been associated with adverse mental health outcomes, increased rates of hypertension, coronary heart disease, and stroke (Halliday et al., 2021). As such, the intersectional theoretical turn within the wider health inequalities literature would benefit from considering place identity as an additional aspect of social identity with consequences for both how other aspects of identity are experienced and for health disparities.

## 2 Conclusion

This article has engaged with the ‘intersectional turn’ in health inequalities research, drawing out the benefits to- and potential contributions of-a more geographical perspective. It has argued that geographical research into health inequalities should begin to more explicitly apply an intersectional lens; and that, place also needs to be considered as an aspect of intersectionality by the wider health inequalities research community. It has suggested ways whereby research into geographical inequalities in health could take up the intersectionality challenge and how in turn, the wider research field could benefit from considering place as an additional aspect of intersectionality. Taken together these provide promising and exciting avenues for the theoretical and empirical development of the field.
